# An antibody microarray analysis of serum cytokines in neurodegenerative Parkinsonian syndromes

**DOI:** 10.1186/1477-5956-10-71

**Published:** 2012-11-23

**Authors:** Philipp Mahlknecht, Sylvia Stemberger, Fabienne Sprenger, Johannes Rainer, Eva Hametner, Rudolf Kirchmair, Christoph Grabmer, Christoph Scherfler, Gregor K Wenning, Klaus Seppi, Werner Poewe, Markus Reindl

**Affiliations:** 1 Clinical Department of Neurology, Innsbruck Medical University, Innsbruck, Austria; 2 Division of Molecular Pathophysiology, Biocenter, Innsbruck Medical University, and Tyrolean Cancer Research Institute, Innsbruck, Austria; 3 Department of Internal Medicine I, Innsbruck Medical University, Innsbruck, Austria; 4 Central Institute for Blood Transfusion and Division for Immunology, University Hospital Innsbruck, Innsbruck, Austria

## Abstract

**Background:**

Microarray technology may offer a new opportunity to gain insight into disease-specific global protein expression profiles. The present study was performed to apply a serum antibody microarray to screen for differentially regulated cytokines in Parkinson's disease (PD), multiple system atrophy (MSA), progressive supranuclear palsy (PSP) and corticobasal syndrome (CBS).

**Results:**

Serum samples were obtained from patients with clinical diagnoses of PD (n = 117), MSA (n = 31) and PSP/CBS (n = 38) and 99 controls. Cytokine profiles of sera from patients and controls were analyzed with a semiquantitative human antibody array for 174 cytokines and the expression of 12 cytokines was found to be significantly altered. In a next step, results from the microarray experiment were individually validated by different immunoassays. Immunoassay validation confirmed a significant increase of median PDGF-BB levels in patients with PSP/CBS, MSA and PD and a decrease of median prolactin levels in PD. However, neither PDGF-BB nor prolactin were specific biomarkers to discriminate PSP/CBS, MSA, PD and controls.

**Conclusions:**

In our unbiased cytokine array based screening approach and validation by a different immunoassay only two of 174 cytokines were significantly altered between patients and controls.

## Background

In addition to the clinical assessment, biomarkers could provide important information not only for the diagnosis of Parkinson’s disease (PD), but also to differentiate idiopathic PD from different entities of atypical Parkinsonian disorders (APDs) as well as to identify persons being at risk of developing the disease. Furthermore, biomarkers could be a helpful tool to evaluate the progression and the severity of PD.

Although several specific biomarker assays in biological fluids such as cerebrospinal fluid (CSF), plasma, urine and serum of patients with neurodegenerative diseases have been under investigation, the vast majority of them have produced disappointing results [1,2]. Recent research focused on the quantification of alpha-synuclein and DJ-1, two proteins critically involved in PD pathogenesis, in CSF with more promising results [3-5]. A comparable problem is seen in Alzheimer’s disease (AD), which is also difficult to diagnose in its earliest stages and CSF biomarkers have been established for the diagnosis of AD [6]. However, peripheral blood is much easier to obtain and not all patients are willing to undergo lumbar puncture. Therefore, it was a major breakthrough when two recent studies described a cytokine-array based investigation of secreted signaling proteins in the peripheral blood that distinguished samples from patients with AD and control subjects [7,8]. Subsequent studies, however, showed controversial results [9,10]. Using a similar technique as a screening tool, another recent study found low epidermal growth factor levels in cognitively impaired PD patients [11]. Increasing evidence has linked chronic central and peripheral immune and inflammatory mechanisms to PD pathogenesis [12] and the pathological processes leading to PD could cause characteristic changes in the concentrations of signaling proteins in the blood. Indeed different cytokines (brain derived neurotrophic factor, tumor necrosis factor alpha and interleukin 6) have been reported to be significantly altered in the sera of PD patients [13-15]. The present study was aimed to apply a screening approach using a cytokine-array with 174 secreted signaling proteins to screen serum samples from patients with PD and from patients with APDs such as progressive supranuclear palsy (PSP), corticobasal syndrome (CBS) and multisystem atrophy (MSA) as well as controls for deregulation of serum proteins. In a second step results from the microarray screening experiment were evaluated by different immunoassays.

## Results

### Human cytokine antibody array experiments

**Figure 1:**
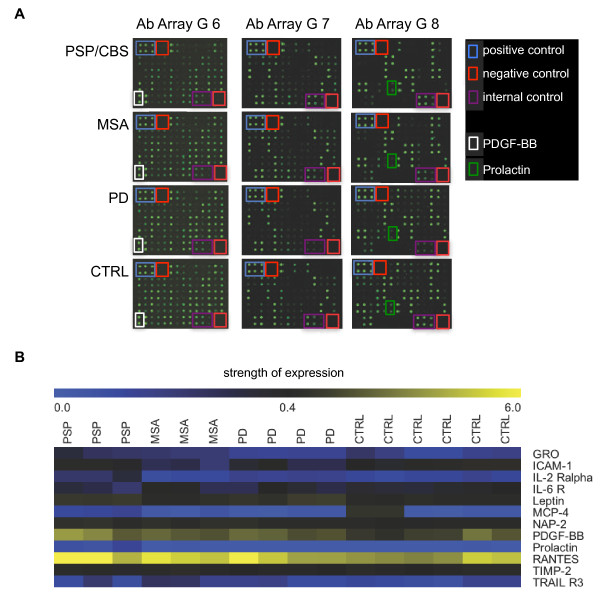
**Identification of serum biomarkers for the discrimination of movement disorders by antibody arrays in the screening cohort.** (**A**) Representative picture of Raybiotech human cytokine antibody array showing the reactivity of pooled serum samples (10 PSP/CBS, 10 MSA, 20 PD and 30 controls) to arrays G series 2000 6, 7 and 8 (174 cytokines). Each protein was measured in duplicates. Signals were scanned with a GenePix 4000B scanner. Blue boxes: positive controls (upper left corner, high intense spots). Red box, negative controls (upper left and lower right corner, no spots). Purple boxes, internal controls IC1, IC and IC3 (lower right corner, spots with three different intensities). White and green colored boxes indicate the location of the detection of two proteins that were significantly different in both the microarray and validation experiment (white = PDGF-BB and green = prolactin). (**B**) Normalized array data of the 174 cytokines were analyzed by SAM to detect differences in their concentrations between pooled serum samples (PSP/CBS, one pool of 10 samples with three replicates; MSA, one pool of 10 samples with three replicates each; PD, two pools of 10 samples with two replicates each; and controls, three pools of 10 samples with two replicates each). The relative concentrations of the 12 cytokines that obtained a significant score (q-value <0.001%) are shown in a “heatmap”. Low concentrations are shown in blue, median concentrations in black and high concentrations in yellow.

We have applied a screening approach with human cytokine antibody arrays using pooled serum samples from patients with PSP/CBS, MSA, PD and age and sex-matched controls (CTRL) to identify putative serum biomarkers for these diseases (Table 1A). In order to exclude an effect of age-related non-neurological diseases on serum cytokine levels we have not only included 63 age and sex matched healthy blood donors (HC), but also 36 patients with internal diseases (INC) in our control group. Figure 1 shows a heatmap of the microarrays for the four groups of patients and controls.

**Table 1:** Demographic and clinical data of patients and controls

**A**. **Screening cohort analyzed in cytokine microarray experiment**					
	**PSP**/**CBS**	**MSA**	**PD**	**CTRL**	**p**-**value**
Number	10	10	20	30	
Female, n(%)	6(60)	6(60)	12(60)	18(60)	ns^b^
Age, y^a^	68(54–75)	66(54–75)	67(52–81)	65(56–80)	ns^c^
Duration, y^a^	4(1–8)	5(1–9)	6(1–37)		ns^c^
Hoehn&Yahr^a^	3(2–5)	4(2–5)	2(1–4)		<0.001^c^
Dementia, n(%)	3(30)	2(20)	2(10)		ns^b^
Treatment, n(%)	9(90)	5(50)	18(90)		ns
	**PSP/CBS**	**MSA**	**PD**	**CTRL**	**p-value**
Number	28	21	97	69	
Female, n(%)	14(50)	14(67)	32(33)	29(42)	0.03^b^
Age, y^a^	74(57–84)	63(46–78)	66(41–85)	63(42–93)	<0.001^c^
Duration, y^a^	3(1–11)	3(1–6)	4(0–27)		ns^c^
Hoehn&Yahr^a^	4(2–5)	4(0–5)	2(0–5)		<0.001^c^
Dementia, n(%)	14(52)	0(0)	10(11)		<0.001^b^
Treatment, n(%)	19(68)	15(71)	63(65)		ns^b^

y = years, ns = not statistically significant, treatment = patients treated with levodopa and/or dopamine agonists and/or monoamine oxidase inhibitors

^a^ data are shown as median (range), p-value: groups were compared using ^b^ Chi-Square test and ^c^ Kruskal-Wallis test.

Using significance of microarray (SAM) analysis, we discovered 12 significant results at a false discovery rate (FDR) cut-off of <0.001%. Figure 1B and Additional file 1 summarize the differentially regulated cytokines found in our arrays using SAM analysis. The significantly differentially expressed cytokines were growth-regulated alpha protein (GRO = CXCL1), intercellular adhesion molecule 1 (ICAM-1 = ICAM1), interleukin-2 receptor alpha chain (IL-2R alpha = IL2RA), interleukin-6 receptor subunit alpha (IL-6 R = IL6R), leptin (LEP), C-C motif chemokine 13 (MCP-4 = CCL13), neutrophil-activating peptide 2 (NAP-2 = PPBP), platelet-derived growth factor subunit B (PDGF-BB = PDGFB), prolactin (PRL), C-C motif chemokine 5 (RANTES = CCL5), metalloproteinase inhibitor 2 (TIMP-2 = TIMP2) and tumor necrosis factor related apoptosis inducing ligand receptor 3 (TRAIL R3 = TNFRSF10C). A post-hoc analysis revealed no statistically significant differences for these cytokines between INC and HC within the control group.

The DAVID v6.7 tool was used for functional annotation clustering of the 12 identified cytokines. As can be seen from Table 2, gene functional classification clustered cytokines into 10 groups with highest stringency with enrichment scores ranging from 1.63 to 7.28. The highest enrichment scores were seen for cytokines associated with immune responses, chemotaxis and cell migration, whereas no association with neuronal or glial function was found.

**Table 2:** Functional annotation clustering of identified cytokines using the DAVID database

**DAVID annotation cluster**	**Enrichment score**, **p**-**value**	**Associated Cytokines**
1. Immune response, immunity and defense	7.28, p < 10^-6^	GRO, ICAM-1, MCP-4, IL-2RA, NAP-2, IL-6R, RANTES
2. Chemotaxis, taxis, locomotory behavior	6.64, p < 10^-8^	GRO, MCP-4, NAP-2, PDGF-BB, IL-6R, RANTES
3. Chemokine	4.23, p < 10^-6^	GRO, MCP-4, NAP-2, RANTES
4. Cell migration, leukocyte migration, cell motility	3.13, p < 10^-5^	ICAM-1, PDGF-BB, IL-6R, RANTES
5. Cell chemotaxis, response to steroid hormone stimulus	2.70, p < 10^-4^	PDGF-BB, IL-6R, RANTES
6. Regulation of cell migration, motion and locomotion	2.31, p < 0.01	ICAM-1, PDGF-BB, IL-6R
7. Response to hormone stimulus	2.24, p < 0.01	leptin, PDGF-BB, IL-6R, RANTES
8. Positive regulation of signal transduction	1.77, p < 0.05	leptin, IL6-R, prolactin
9. Diabetes type 2, metabolic disease, reproduction	1.63, p < 0.05	leptin, ICAM-1, IL-6R, RANTES

### Validation of microarray data by ELISA and flow-cytomix assays

**Figure 2:**
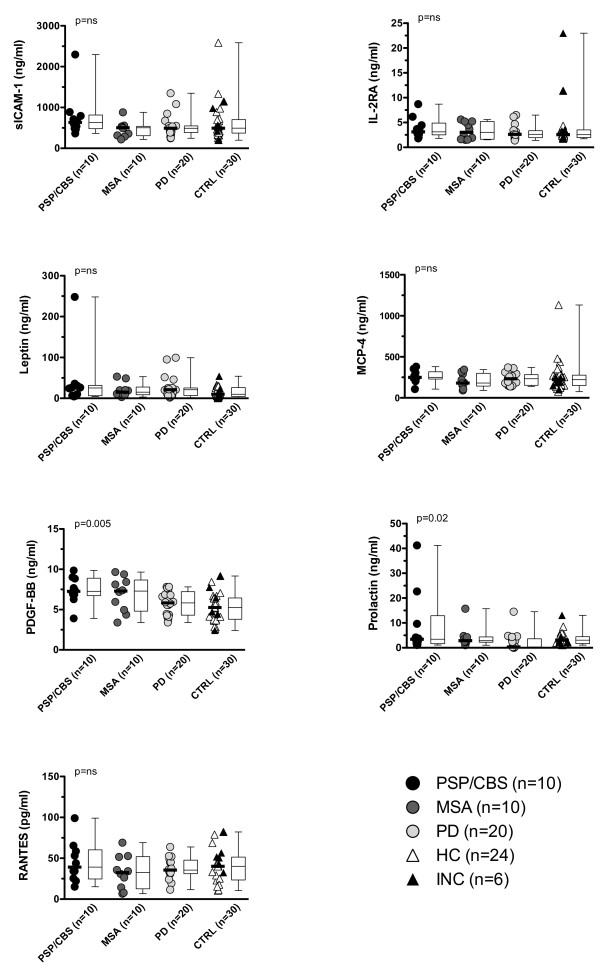
**Validation of seven differentially expressed cytokines in patients with PSP/CBS, MSA and controls in the screening cohort.** Individual data points are shown as circles or triangles and horizontal bars indicate medians. In addition data are shown as box plots with medians indicated as horizontal bars with boxes. Groups were compared using the Kruskal-Wallis test and Dunn’s multiple comparison post-hoc test and overall p-values for comparison of PSP/CBS, MSA and combined controls or for comparison of PSP/CBS, MSA, HC and INC are shown in each figure. Ns = statistically not significant.

Since the cytokine microarray analysis was performed using pooled samples from the screening cohort, we decided to validate the results of the microarray analysis by ELISA (MCP-4, prolactin, RANTES and IL-2RA) and by Flow-Cytomix assays (ICAM-1, leptin and PDGF-BB) applied on individual samples for seven of the 12 cytokines. These seven cytokines were selected based on the results from the microarray experiments, the availability of commercial available test kits and the amount of serum left. We used the same serum samples as shown in Table 1A and the results of these validation experiments are shown in Table 3 and Figure 2. However, only two (PDGF-BB and prolactin) of the seven cytokines were significantly different amongst patients (PSP/CBS, MSA and PD) and controls, whereas we could not confirm the cytokine microarray results for ICAM-1, IL-2RA, leptin, MCP-4 and RANTES (Figure 2). A separate analysis of both control groups, HC and INC, did not change the results.

**Table 3:** Validation of the serum cytokine array experiment in the initial screening cohort

**Cytokine**	**Method**	**PSP**/**CBS**	**MSA**	**PD**	**Controls**	**p**-**value**
ICAM-1	Array	0.98 ± 0.25	0.47 ± 0.09	0.62 ± 0.15	1.00 ± 0.28	<0.05^a^
(ICAM1)	FCM	1.25 ± 0.85	0.75 ± 0.29	0.86 ± 0.43	1.00 ± 0.73	ns^b^
IL-2 Ra	Array	2.14 ± 0.47	1.87 ± 0.10	1.24 ± 0.06	1.00 ± 0.14	<0.05^a^
(IL2RA)	ELISA	1.44 ± 0.76	1.24 ± 0.61	1.15 ± 0.55	1.00 ± 0.29	ns^b^
Leptin	Array	1.93 ± 0.11	1.13 ± 0.23	1.91 ± 0.46	1.00 ± 0.11	<0.05^a^
(LEP)	FCM	2.95 ± 4.89	1.42 ± 1.15	1.86 ± 1.88	1.00 ± 0.89	ns^b^
MCP-4	Array	7.18 ± 0.85	2.16 ± 1.39	1.71 ± 0.80	1.00 ± 0.76	<0.05^a^
(CCL13)	ELISA	1.15 ± 0.32	0.91 ± 0.37	1.02 ± 0.32	1.00 ± 0.42	ns^b^
PDGF-BB	Array	1.87 ± 0.50	1.34 ± 0.18	1.34 ± 0.22	1.00 ± 0.44	<0.05^a^
(PDGFB)	FCM	1.42 ± 0.30	1.32 ± 0.39	1.11 ± 0.28	1.00 ± 0.33	<0.05^b^
Prolactin	Array	4.17 ± 1.74	1.03 ± 0.15	0.99 ± 0.08	1.00 ± 0.19	<0.05^a^
(PRL)	ELISA	3.06 ± 4.15	1.39 ± 1.34	0.71 ± 1.12	1.00 ± 0.50	<0.05^b^
RANTES	Array	1.46 ± 0.29	1.32 ± 0.11	1.20 ± 0.25	1.00 ± 0.20	<0.05^a^
(CCL5)	ELISA	1.13 ± 0.59	0.83 ±0.49	0.94 ± 0.32	1.00 ± 0.51	ns^b^

Cytokine microarray data were validated by ELISA (MCP-4, prolactin, RANTES and IL-2RA) or by Flow-Cytomix assays (ICAM-1, Leptin and PDGF-BB) in individual samples. All data are shown as means with standard deviations of the ratio to the mean of the control group. Array = Raybiotech cytokine microarray, FCM = Flow-Cytomix, ELISA = enzyme-linked immunosorbent assay, ns = statistically non significant.

P-value: groups were compared using ^a^ SAM analysis and ^b^ Kruskal-Wallis test.

**Figure 3:**
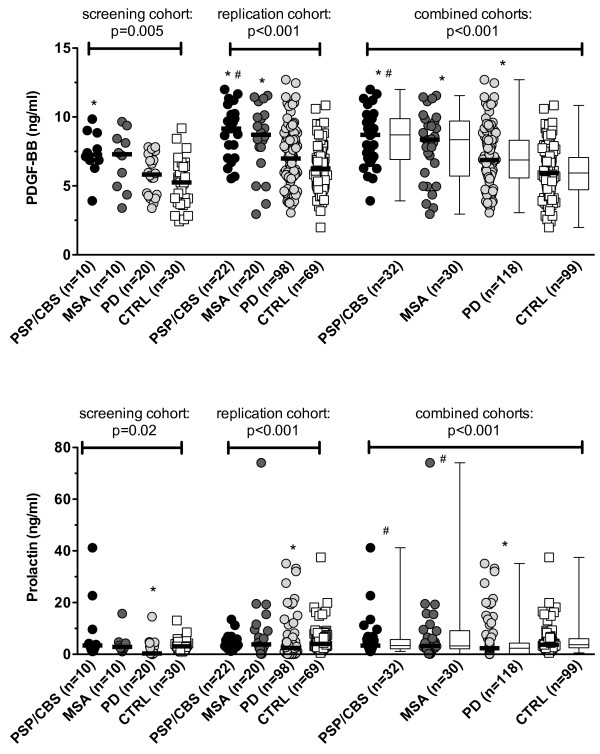
**Differential expression of PDGF-BB and prolactin in patients with PSP/CBS, MSA and controls in the screening and replication cohorts.** Individual data points are shown as circles and horizontal bars indicate medians. In addition data are shown as box plot with medians indicated as horizontal bars with boxes. Groups were compared using the Kruskal-Wallis test and Dunn’s multiple comparison post-hoc test and overall p-values are shown in each figure. * Significant differences to the control group, # significant differences to PD patients.

In a next step we extended the analysis of PDGF-BB and prolactin to a replication cohort of patients and controls (Table 1B) and to all patients and controls. Figures 2 and 3 demonstrate that these two cytokines were significantly different among the four groups in the screening and replication cohorts and in the combined data. PDGF-BB was significantly increased in PSP/CBS, MSA and PD and prolactin was significantly decreased in PD (Figure 3, Table 4). From Table 4 it is also evident that PDGF-BB levels were mainly influenced by the clinical diagnosis, whereas prolactin levels were more strongly influenced by antiparkinson treatment (p = 7x10^-13^) than by clinical diagnosis (p = 7x10^-7^).

**Table 4:** Association of serum PDGF-BB and Prolactin levels (in ng/ml) with clinical parameters

	**PDGF**-**BB(ng/ml)**	**Prolactin(ng/ml)**
Disease:		
PSP/CBS (n = 38) ^a^	8.7 (2.7-12.6)	3.4 (0.3-41.2)
MSA (n = 31) ^a^	8.4 (2.9-11.6)	3.3 (0.1-74.1)
PD (n = 117) ^a^	6.9 (3.0-12.7)	2.3 (0.0-35.1)
Controls (n = 99) ^a^	5.9 (2.0-10.8)	3.7 (0.5-37.5)
	p = 4x10^-7 b^	p = 3x10^-7 b^
Females (n = 132) ^a^	6.7 (2.4-12.7)	3.1 (0.0-74.1)
Males (n = 153) ^a^	6.8 (2.0-12.0)	3.3 (0.0-37.4)
	p = ns ^c^	p = ns ^c^
Age, y	R = 0.006	R = −0.040
	p = ns ^d^	p = ns ^d^
Duration, y	R = −0.183	R = −0.226
	p = ns ^d^	p = ns ^d^
Hoehn&Yahr	R = 0.105	R = 0.217
	p = ns ^e^	p = 0.02 ^e^
Dementia (n = 31) ^a^	8.3 (4.2-12.6)	3.1 (0.0-35.1)
No Dementia (n = 148) ^a^	7.1 (2.6-12.7)	2.7 (0.0-74.1)
	p = 0.038 ^c^	p = ns ^c^
Parkinson treatment:		
None (n = 48) ^a^	6.5 (2.0-12.7)	3.6 (0.2-37.5)
LD (n = 48) ^a^	7.4 (2.7-12.6)	4.0 (0.0-74.1)
DA (n = 8) ^a^	6.6 (3.7-8.6)	0.0 (0.0-3.1)
MAO-B Inh. (n = 8) ^a^	8.1 (5.5-11.4)	2.8 (1.7-4.7)
LD + DA (n = 48) ^a^	6.1 (2.9-11.4)	0.2 (0.0-33.2)
LD + MAO-B Inh. (n = 12) ^a^	10.3 (5.0-11.7)	9.4 (0.3-41.2)
LD + DA + MAO-B Inh. (n = 7) ^a^	6.9 (5.6-10.4)	0.3 (0.0-19.9)
	p = 0.002 ^b^	p = 7x10^-13 b^

^a^ Data are shown as median (range). Associations were statistically analyzed by ^b^ Kruskal Wallis test, ^c^ Mann–Whitney U tests, ^d^ linear regressions and ^e^ Spearman’s correlations. P-values were corrected for 7 comparisons by Bonferroni’s correction for multiple comparisons.

Abbreviations: ns = not statistically significant, R = correlation coefficient, LD = patients treated with levodopa, DA = patients treated with dopmaine agonists, MAO-B Inh. = patients treated with monoamine oxidase inhibitors.

This was also seen in a multivariate analysis including prolactin serum levels as dependent variable and diagnoses (PSP/CBS, MSA, PD, controls), sex, age and dopaminergic antiparkinsonian treatment as independent variables revealed that serum prolactin levels were influenced only by antiparkinsonian treatment (p < 0.001), but not by diagnoses, sex and age (p = ns). Furthermore from Additional file 2 it is evident that in the subgroup of early untreated patients median prolactin levels did not differ between the various patient groups [PSP/CBS (n = 10; 3.3 ng/ml; range 1.1-5.8), MSA (n = 11; median 2.9 ng/ml; 0.2-19.3), PD (n = 37; median 3.6 ng/ml; 0.2-21.5) and controls (n = 99; median 3.7 ng/ml; 0.5-37.5)].

In contrast, multivariate analysis revealed that serum PDGF-BB levels were influenced by the diagnoses only (p < 0.001), but not by antiparkinsonian treatment, sex and age (p = ns). Also in our early untreated patients PDGF-BB serum levels were significantly different (p < 0.001) between patient groups (Additional file 2). Median PDGF-BB serum levels were 8.6 ng/ml (range 5.7-12.0) in patients with PSP/CBS (n = 10), 8.4 ng/ml (4.4-10.1) in patients with MSA (n = 11), 7.9 ng/ml (3.0-12.7) in patients with PD (n = 37) and 5.9 ng/ml (2.0-10.8) in controls (n = 99).

## Discussion

To our knowledge this is the first study using an unbiased cytokine microarray analysis approach to identify potential serum biomarkers for the discrimination of neurodegenerative parkinsonian disorders. In our study the primary screen using cytokine microarray analysis on pooled samples yielded 12 cytokines differentially regulated in PSP/CBS, MSA, PD and controls (GRO, ICAM-1, IL-2 R-alpha, IL-6 R, leptin, MCP-4, NAP-2, PDGF-BB, prolactin, RANTES, TIMP-2 and TRAIL R3). Functional annotation clustering revealed that these cytokines are associated with immune responses, chemotaxis and cell migration, whereas no association with neuronal or glial function was found. These results suggest that it is rather unlikely that the identified cytokines are markers reflecting specific pathophysiological processes for neurodegenerative parkinsonism.

By using a second independent, analytic method (ELISA or bead-based immunoassays), we have tried to confirm the results derived from the microarray analysis in the same samples for seven of these 12 cytokines (ICAM-1, IL-2 RA, leptin, MCP-4, PDGF-BB, prolactin and RANTES). However, only two (PDGF-BB and prolactin) of the seven cytokines were significantly different amongst patient groups (PSP/CBS, MSA and PD) and controls using both methods. In a second step we were able to confirm these results in a different cohort containing larger number of patients and controls. The striking difference between initial analysis and initial replication by a different method likely reflects differences in the sensitivity and specificity of the used antibodies and appears to be consistent with the frequent irreproducibility of many serum biomarker studies of neurodegenerative diseases. For instance the results of a recent study describing a cytokine-array based investigation of protein-panels enabling to distinguish patients with AD from HC [7] could not be reproduced in two subsequent studies [9,10]. Beside this methodological concern, our results suggest that the serum prolactin levels are influenced by dopaminergic antiparkinsonian treatment, but not the patient group; dopaminergic antiparkinsonian treatment remained the only significant variable on prolactin levels in a multivariate analysis and patients with no such treatment had similar serum prolactin levels to controls. This is in line with studies indicating a crucial role for dopamine as an inhibitor of prolactin production as well as with studies suggesting that untreated PD patients have normal prolactin release, whereas pharmacologic stimulation of dopamine D2-receptors with dopaminergic antiparkinsonian treatment leads to decreased serum prolactin levels [16-18], corroborating the reliability of our cytokine-array screening approach.

Serum PDGF-BB levels were significantly increased in the patient groups compared to the controls with the highest levels found in PSP/CBS. PDGF-BB, a member of the platelet-derived growth factor family, is a homodimer encoded by the PDGFB gene [19]. PDGF was originally discovered in serum and identified as a major mitogenic factor for connective tissue cells as well as some epithelial and endothelial cells. In addition, PDGF is chemotactic for fibroblasts, smooth muscle cells, neutrophils and mononuclear cells. However, PDGF also appears to be ubiquitous in neurons throughout the CNS, where it is suggested to play an important role in neural development, function and neuron survival as well as in mediation of glial cell proliferation and differentiation [19]. Experimental studies from the 1990s demonstrated that PDGF-BB acts as a trophic factor for rat and human mesencephalic dopaminergic neurons promoting gene expression, survival and neurite outgrowth in culture [20,21]. In the 6-OHDA rat model, PDGF could counteract the 6-OHDA-induced degeneration of mesencephalic DA neurons when administered prior to the insult [22]. In the same in vivo model, PDGF-BB as well as BDNF administration post insult was capable of increasing the numbers of newly formed cells in the striatum and substantia nigra [23]. To the best of our knowledge there are no studies reporting on PDGF concentrations in brain tissue or in the CSF from parkinsonian patients. In peripheral blood, levels of PDGF-BB have been analyzed in AD, but the results of studies were controversial [7,9,10,24]. Interestingly, the most recent of these studies also included 11 demented PD patients and, in line with our results, they found an increase in PDGF-BB levels in their plasma [10].

In our cohort of patients with neurodegenerative parkinsonian syndromes, there was no association of PDGF-BB levels with the disease duration or the Hoehn and Yahr score. Also in the subgroups of early untreated patients PDGF was elevated to the same extent as in the whole groups. Thus, it is tempting to speculate that the increased serum PDGF-BB levels might reflect early compensatory mechanisms as a response to neurodegeneration. This would appear consistent with increasing evidence that immunological and inflammatory processes including microglial over-activation as well as increased synthesis and release of cytokines could be a key player in PD pathogenesis [12]. PDGF-BB elevations could therefore represent an important factor in central and peripheral communication between neurons, glial cells and peripheral immune cells. Besides the expression in neurons and Schwann cells, PDGF-BB is also synthesized by vascular endothelial cells, macrophages, fibroblasts and megakaryocytes [25]. Since PDGF-BB has several important functions in the peripheral circulation such as mitogenic and chemotactic effects on mesenchymal stem cells [26-29], it is more likely that the increased serum PDGF-BB levels observed in our study might reflect a response to pathological changes in the periphery. This could explain why PDGF-BB was also detectable in our control sera to a marked extent, which accounts for the suboptimal differentiation of neurodegenerative Parkinsonian syndromes from controls. Our study has some limitations: it was performed in patients with a clinical diagnosis of neurodegenerative parkinsonian syndromes without pathological confirmation. Hence, misdiagnosis in some patients, especially in the early disease stages, cannot be excluded. Also, some of the included PSP patients suffered from the most reliably identifiable classic picture of PSP (i.e. Richardson’s syndrome), whereas the true diagnostic dilemma lies with atypical presentations like PSP-parkinsonism [30]. Given the pathological heterogeneity of a ‘corticobasal syndrome’ [31], most commonly including CBS and other neurodegenerative causes such as PSP with both diseases sharing the same tauopathy and due to the limited number of CBS patients (n = 8) included into the present study, these two groups were gathered together. However, in all validation experiments CBS and PSP patients were analyzed separately and we found no differences between these groups. Furthermore, we only analyzed seven out of twelve deregulated cytokines in the initial cytokine array, depending on the availability of commercial available test kits. Therefore, the deregulation of five proteins significantly altered in the initial screening was not further validated (GRO, IL-6 R = IL6R, NAP-2, TIMP-2, TRAIL R3). The cross sectional design of our study did not allow for a direct correlation of PDGF-BB levels and disease progression. Therefore a longitudinal study is now needed to address this important question. Finally, our control group included not only healthy controls, but also patients with internal diseases. We think that this is not a limitation but rather a strength since these controls could avoid confounding effects of internal diseases related to aging in patients with neurodegenerative diseases. However, a post-hoc analysis revealed no differences between INC and HC for the cytokines analyzed in this study.

## Conclusions

In conclusion we have for the first time used a serum cytokine microarray approach to identify factors deregulated in PSP/CBS, MSA and PD. Only two of 174 cytokines analyzed (PDGF-BB and prolactin) were significantly altered between patients and controls, but none of them seem to be useful biomarkers for these diseases.

## Methods

### Ethics statement

The present study was approved by the ethical committee of Innsbruck Medical University (study no.: AM1979d) and all patients gave written informed consent to the study protocol.

### Patients and serum samples

Patients with PSP or CBS (n = 38), PD (n = 117) and MSA (n = 31) were seen at the Movement Disorder outpatient clinic at the Clinical Department of Neurology at Innsbruck Medical University. Clinical diagnosis of these disorders had been made according to established criteria by movement disorder specialists (K.S., C.S., G.K.W., W.P.) and most patients were under regular follow up for more than 5 years at our institution. Due to the limited number of CBS patients (n = 8) included into the present study and due to the clinical and pathological overlap between PSP and CBS [31], these two groups were analysed together. Dementia was clinically diagnosed using DSM-IV criteria.

Serum samples of 63 age and gender matched healthy blood donors (HC) obtained from the blood transfusion center of Innsbruck University Hospital and 36 patients with internal diseases (INC; two or more of the following: chronic kidney disease, coronary heart disease, hypertonus, type 2 diabetes, chronic obstructive pulmonary disease, liver cirrhosis, autoimmune disease, infectious diseases and malignancy) without neurological impairment recruited from the Department of Internal Medicine were used as controls. The latter control group was chosen to control for a confounding effect of internal diseases related to disability and/or aging.

All serum samples were collected prospectively from 2007 to 2010, after lunch (1.00 pm to 4.00 pm during outpatient clinic and ward rounds) respectively, centrifuged and stored at −80°C within one hour after blood withdrawal. The clinical and demographic data of all patients are shown in Table 1. Before analysis sample were aliquoted and stored at −80°C (one freeze-thaw cycle).

### Human cytokine antibody array and immunoassays

Human sera of patients and control groups were pooled (Table 1) and their cytokine profiles were analyzed with a semiquantitative human cytokine antibody array that detects 174 cytokines in one experiment (RayBio Human Cytokine Antibody Array G series 2000; Raybiotech, Norcross GA, USA; http://www.raybiotech.com/G_Series.asp; July 2012). The array consisted of three glass slides (array 6, 7, and 8) that were pretreated according to the manufacturer's instructions and incubated with 2-fold diluted serum pools for 2 hours. All sample measurements were performed in duplicate. The array glass slides were washed, incubated with a biotin-conjugated anti-cytokine mix for 2 hours, washed again, and developed for 2 hours with Cy3-conjugated streptavidin. The signals were scanned with a GenePix 4000B scanner (Axon Instruments, GenePix version 5.0) and analyzed with the Raybiotech analysis tool, a data analysis program based on Microsoft Excel technology specifically designed to analyze Raybiotech Antibody Array G Series. Signals were normalized using internal, positive and negative controls included on the array. All data is MIAME compliant and raw and normalized cytokine microarray data have been deposited to the Gene Expression Omnibus (GEO) database, Series GSE32041 (accession numbers GSE32037, GSE32039, GSE32040 and GSE32041).

### Determination of cytokine levels by ELISA and fluorescence bead-based assays

Serum cytokine and growth factor levels were measured by ELISA (MCP-4, prolactin, RANTES and sIL-2R) or by fluorescence bead-based assays (ICAM-1, leptin and PDGF-BB), respectively. ELISA kits specific for human MCP-4 (DY327) and prolactin (DY682) were purchased from R&D Systems (Minneapolis, MN, USA). ELISA kits specific for human RANTES (BMS287/2INST) and IL-2RA (BMS212INSTCE) were purchased from eBioscience (Vienna, Austria). Fluorescence bead-based assays (Flow Cytomix) for human sICAM-1 (BMS80201FF), Leptin (BMS82039/2FF) and PDGF-BB (BMS82071FF) were obtained from Bender Med Systems.

All analyses were performed according to the manufacturer guidelines. Serum dilutions were 1:2 for MCP-4, 1:10 for prolactin, 1:15 for RANTES, 1:15 for IL-2RA, 1:1 for ICAM-1, 1:1 for IL-6, 1:1 for leptin and 1:1 for PDGF-BB. Technical details and performance of the assays used are shown in Additional file 3.

### Statistical analysis and bioinformatics

Micorarray data were statistically analyzed with the TIGR MeV_4_5 (Multiple Experiment Viewer),) Java tool for genomic data analysis (http://mev-tm4.sourceforge.net/; July 2012) [32], using the significance analysis of micoarrays (SAM) method. Multi-class SAM was used to identify significant cytokines based on differential expression between the four groups at a false discovery rate (FDR, expected proportion of false positives among rejected hypotheses) of 0%.

Statistical analysis for the validation experiments (means, medians, range, standard deviations, significance of group differences and linear regression) were evaluated using IBM SPSS software (release 18.0, SPSS Inc., USA) or GraphPad Prism 5 (GraphPad, San Diego, USA). Between-group comparisons were performed with Kruskal-Wallis test, Dunn’s multiple comparison post-hoc test, Mann–Whitney *U* test, Fisher’s exact test, Chi-square test and multivariate analysis. Correlation of parameters was analyzed with Spearman’s non-parametric correlation or linear regression analysis. Statistical significance was defined as two-sided p-value < 0.05 and Bonferroni corrections were applied for multiple comparisons when appropriate.

The Database for Annotation, Visualization and Integrated Discovery (DAVID) v6.7 was used for functional annotation clustering of identified cytokines (http://david.abcc.ncifcrf.gov/) [33].

## Competing interests

The authors declare that they have no competing interests.

## Authors’ contributions

PM, KS, WP and MR were responsible for planning and designing the study. FS, EH, RK, CG, CS, GKW, KS and WP collected samples and clinical data. PM, SS, FS and JR performed the experiments. PM, JR and MR performed the statistical analysis. PM, JR, KS, WP and MR wrote the manuscript. All authors read and approved the final manuscript.

## Supplementary Material

Additional file 1 Results of cytokine microarray experiment. Click here for file

Additional file 2 Differential expression of PDGF-BB and prolactin in patients with PSP/CBS, MSA and controls without anti-Parkinsonian treatment. Click here for file

Additional file 3 Technical characteristics of the immunoassays used for validation. Click here for file
