# Supplementary material for: An antibody microarray analysis of serum cytokines in neurodegenerative Parkinsonian syndromes
Source: Proteome Sci. 2012 Nov 23;10:71. doi: 10.1186/1477-5956-10-71 (PMC3539904; doi:10.1186/1477-5956-10-71)
Supplement: Additional file 1 — Results of cytokine microarray experiment. [file 1477-5956-10-71-S1.docx]

### Additional file 1

### Results of cytokine microarray experiment

**The q-value approach implemented in SAM was used to estimate false discovery rates.**

| **Cytokine Name** | **Expected *d*-value** | **Observed *d*-value** | **FDR (%)** | **Official Symbol** | **Full Name** | **Genbank accession** |
| --- | --- | --- | --- | --- | --- | --- |
| GRO | 0.204 | 1.303 | 0.000 | CXCL1 | Growth-regulated alpha protein | NM_001511 |
| ICAM-1 | 0.218 | 1.207 | 0.000 | ICAM1 | Intercellular adhesion molecule 1 | NM_000201.2 |
| IL-2 Ralpha | 0.451 | 2.241 | 0.000 | IL2RA | Interleukin 2 receptor alpha chain | NM_000417 |
| IL-6 R | 0.259 | 1.245 | 0.000 | IL6R | Interleukin 6 receptor subunit alpha | NM_000565 |
| Leptin | 0.000 | 1.923 | 0.000 | LEP | Leptin | NM_000230 |
| MCP-4 | 0.000 | 2.038 | 0.000 | CCL13 | C-C motif chemokine 13 | NM_005408 |
| NAP-2 | 0.084 | 1.546 | 0.000 | PPBP | Neutrophil-activating peptide 2 | NM_002704 |
| PDGF-BB | 0.100 | 1.324 | 0.000 | PDGFB | Platelet-derived growth factor subunit B | NM_002608 |
| Prolactin | 0.650 | 1.503 | 0.000 | PRL | Prolactin | NM_000948 |
| RANTES | 0.106 | 1.299 | 0.000 | CCL5 | C-C motif chemokine 5 | NM_002985 |
| TIMP-2 | 0.326 | 1.279 | 0.000 | TIMP2 | Metalloprotein- ase inhibitor 2 | NM_003255 |
| TRAIL R3 | 0.334 | 1.151 | 0.000 | TNFRSF10C | Tumor necrosis factor receptor superfamily, member 10c, decoy without an intracellular domain | NM_003841.3 |
| Acrp30 | 0.145 | 0.936 | 4.382 | ADIPOQ | Adiponectin | NM_001177800.1 |
| IL-6 | 0.000 | 0.945 | 4.382 | IL6 | Interleukin 6 | NM_000600 |
| EGF-R | 0.179 | 1.012 | 5.008 | ERBB1 | Epidermal growth factor receptor | NM_005228 |
| PARC | 0.094 | 1.123 | 5.393 | CCL18 | C-C motif chemokine 18 | NM_002988 |
| IL-15 | 0.000 | 0.876 | 7.791 | IL15 | Interleukin 15 | NM_000585 |
| TGF-beta 1 | 0.128 | 0.887 | 7.791 | TGFB1 | Transforming growth factor beta-1 | NM_000660 |
| IFN-gamma | 0.000 | 0.822 | 9.145 | IFNG | Interferon gamma | NM_000619 |
| TNF-alpha | 0.137 | 0.817 | 9.145 | TNF | Tumor necrosis factor | NM_000594 |
| Angiogenin | 0.000 | 0.853 | 10.016 | ANG | Angiogenin | NM_001145 |
| MIP-1-delta | 0.000 | 0.847 | 10.016 | CCL15 | C-C motif chemokine 15 | NM_032965 |
| sgp130 | 0.307 | 0.837 | 10.016 | IL6ST | IL6ST Interleukin 6 signal transducer (gp130, oncostatin M receptor)IL6ST interleukin 6 signal transducer (gp130, oncostatin M receptor) | NM_002184.3 |
| uPAR | 0.341 | 0.787 | 11.686 | PLAUR | Urokinase plasminogen activator surface receptor | NM_001005376 |
| IL-1alpha | 0.000 | 0.756 | 13.484 | IL1A | Interleukin-1 alpha | NM_000575 |
| PDGF Rbeta | 0.623 | 0.764 | 13.484 | PDGFRB | Beta-type platelet-derived growth factor receptor | NM_002609 |
| PDGF AA | 0.586 | 0.692 | 19.476 | PDGFA | Platelet-derived growth factor subunit A | NM_002607 |
| SCF R | 0.667 | 0.680 | 20.033 | SCFR | Mast/stem cell growth factor receptor | NM_000222 |
| Eotaxin | 0.000 | 0.643 | 24.879 | CCL11 | Eotaxin | NM_002986 |
| IL-5 | 0.000 | 0.648 | 24.879 | IL5 | Interleukin 5 | NM_000879 |
| IL-7 | 0.000 | 0.640 | 24.879 | IL7 | Interleukin 7 | NM_000880 |
| IGFBP-6 | 0.229 | 0.614 | 25.496 | IGFBP6 | Insulin-like growth factor-binding protein 6 | NM_002178 |
| L-Selectin | 0.519 | 0.626 | 26.293 | SELL | L-selectin | NM_000655 |
| TIMP-1 | 0.322 | 0.605 | 26.809 | TIMP1 | Metalloproteinase inhibitor 1 | NM_003254 |
| MDC | 0.000 | 0.580 | 28.425 | CCL22 | C-C motif chemokine 22 | NM_002990 |
| PDGF-AB | 0.596 | 0.591 | 28.425 | PDGF-AB | Platelet-derived growth factor A/B heterodimer |  |
| TGF-alpha | 0.722 | 0.585 | 28.425 | TGFA | Protransforming growth factor alpha | NM_003236 |
| IL-13 | 0.000 | 0.573 | 28.600 | IL13 | Interleukin 13 | NM_002188 |
| MCP-3 | 0.000 | 0.566 | 28.765 | CCL7 | C-C motif chemokine 7 | NM_006273 |
| ALCAM | 0.359 | 0.527 | 32.954 | ALCAM | CD166 antigen | NM_001627 |
| Axl | 0.158 | 0.527 | 32.954 | UFO | Tyrosine-protein kinase receptor UFO |  |
| BMP-6 | 0.000 | 0.516 | 32.954 | BMP6 | Bone morphogenetic protein 6 | NM_001718 |
| Eotaxin-2 | 0.000 | 0.532 | 32.954 | CCL24 | Eotaxin-2 | NM_002991 |
| GM-CSF | 0.000 | 0.516 | 32.954 | CSF2 | Granulocyte-macrophage colony stimulating factor | NM_000758 |
| IL-10 | 0.000 | 0.515 | 32.954 | IL10 | Interleukin 10 | NM_000572 |
| LAP | 0.495 | 0.514 | 32.954 | TGFb | Latency-associated peptide | NM_000660.4 |
| MMP-9 | 0.553 | 0.534 | 32.954 | MMP9 | Matrix metalloproteinase-9 | NM_004994 |
| Siglec-5 | 0.702 | 0.507 | 32.954 | SIGLEC5 | Sialic acid-binding Ig-like lectin 5 | NP_003821.1 |
| TIMP-4 | 0.848 | 0.513 | 32.954 | TIMP4 | Metalloproteinase inhibitor 4 | NM_003256 |
| TNF-beta | 0.141 | 0.505 | 32.954 | LTA | Tumor necrosis factor beta | NP_000586.2 |
| IL-16 | 0.000 | 0.476 | 36.406 | IL16 | Interleukin 16 | NM_172217 |
| MIP-1beta | 0.280 | 0.474 | 36.406 | CCL4 | C-C motif chemokine 4 | NM_002984 |
| GDNF | 0.000 | 0.469 | 37.042 | GDNF | Glial-derived Neurotrophic Factor | NM_000514 |
| BDNF | 0.000 | 0.429 | 40.230 | BDNF | Brain-derived neurotrophic factor | NM_170735 |
| CTACK | 0.173 | 0.435 | 40.230 | CCL27 | C-C motif chemokine 27 | NM_006664 |
| FGF-7 | 0.000 | 0.441 | 40.230 | FGF7 | Fibroblast growth factor-7 | NM_002009 |
| IL-3 | 0.000 | 0.434 | 40.230 | IL3 | Interleukin 3 | NM_000588 |
| M-CSF | 0.000 | 0.449 | 40.230 | CSF1 | Macrophage colony-stimulating factor 1 | NM_000757 |
| NT-3 | 0.089 | 0.429 | 40.230 | NTF3 | Neurotrophin-3 | NM_002527 |
| TARC | 0.122 | 0.430 | 40.230 | CCL17 | Thymus and activation-regulated chemokine | NP_002978.1 |
| TRAIL R4 | 0.337 | 0.451 | 40.230 | TNFRSF10D | TNFRSF10D tumor necrosis factor receptor superfamily, member 10d, decoy with truncated death domain | NM_003840.3 |
| SCF | 0.112 | 0.421 | 41.843 | SCF | Stem cell factor | NP_000890.1, |
| CXCL- 16 | 0.386 | 0.405 | 43.148 | CXCL16 | C-X-C motif chemokine 16 | NM_022059 |
| M-CSF R | 0.527 | 0.414 | 43.148 | CSF1R | Macrophage colony-stimulating factor 1 receptor | NP_005202.2 |
| NT-4 | 0.291 | 0.406 | 43.148 | NTF4 | Neurotrophin-4 | NM_006179 |
| CD14 | 0.382 | 0.398 | 44.619 | CD14 | Monocyte differentiation antigen CD14 | NP_000582.1. |
| Angiopoietin-2 | 0.152 | 0.356 | 48.541 | ANGPT2 | Angiopoietin-2 | NM_001147.2 |
| E-Selectin | 0.404 | 0.348 | 48.541 | SELE | E-selectin | NM_000450 |
| ENA-78 | 0.181 | 0.384 | 48.541 | CXCL5 | Epithelial neutrophil-activating protein 78 | NM_002994 |
| Eotaxin-3 | 0.000 | 0.361 | 48.541 | CCL26 | Eotaxin-3 | NM_006072 |
| Fas/TNFRSF6 | 0.184 | 0.357 | 48.541 | FAS | Tumor necrosis factor receptor superfamily member 6 | NP_000034.1. |
| HGF | 0.215 | 0.369 | 48.541 | HGF | Hepatocyte growth factor | NM_001010934 |
| ICAM-2 | 0.414 | 0.348 | 48.541 | ICAM2 | Intercellular adhesion molecule 2 | NM_000873 |
| IGF-II | 0.419 | 0.363 | 48.541 | IGF2 | Insulin-like growth factor-2 | NM_000612 |
| IL-1beta | 0.000 | 0.374 | 48.541 | IL1B | Interleukin-1 beta | NM_000576 |
| LIGHT | 0.000 | 0.348 | 48.541 | TNFSF14 | Tumor necrosis factor ligand superfamily member 14 | NM_172014 |
| MIP-3beta | 0.283 | 0.367 | 48.541 | CCL19 | C-C motif chemokine 19 | NM_006274 |
| MMP-1 | 0.534 | 0.385 | 48.541 | MMP1 | Interstitial collagenase | NM_002421 |
| BLC | 0.000 | 0.331 | 50.082 | CXCL13 | B-lymphocyte chemoattractant | NM_006419 |
| Dtk | 0.176 | 0.326 | 50.082 | Dtk | Tyrosine-protein kinase receptor TYRO3 | NP_006284.2 |
| Flt-3 Ligand | 0.000 | 0.323 | 50.082 | FLT3 | Fms-like tyrosine kinase-3 Ligand | NM_004119 |
| IL-4 | 0.000 | 0.335 | 50.082 | IL4 | Interleukin 4 | NM_172348 |
| MIF | 0.273 | 0.324 | 50.082 | MIF | Macrophage migration inhibitory factor | NM_002415 |
| MIP-3-alpha | 0.070 | 0.334 | 50.082 | CCL20 | C-C motif chemokine 20 | NM_004591 |
| VEGF R3 | 1.140 | 0.317 | 50.730 | VEGF R3 | Vascular endothelial growth factor receptor 3 | NP_002011.2 |
| B7-1(CD80) | 0.363 | 0.296 | 54.451 | CD80 | T-lymphocyte activation antigen CD80 | NM_005191 |
| CNTF | 0.000 | 0.290 | 54.451 | CNTF | Ciliary neuronotrophic factor | NM_000614 |
| ErbB3 | 0.399 | 0.278 | 54.451 | ERBB3 | Receptor tyrosine-protein kinase erbB-3 | NP_001005915.1. |
| IGFBP-1 | 0.000 | 0.292 | 54.451 | IGFBP1 | Insulin-like growth factor binding proteins 1 | NM_001013029 |
| IGFBP-4 | 0.000 | 0.280 | 54.451 | IGFBP4 | Insulin-like growth factor binding proteins 4 | NM_001552 |
| IL-1 R4/ST2 | 0.235 | 0.273 | 54.451 | IL1RL1 | Interleukin 1 receptor-like 1 | NP_003847.2, |
| MCP-1 | 0.000 | 0.276 | 54.451 | CCL2 | C-C motif chemokine 2 | NM_002982 |
| SDF-1 | 0.117 | 0.300 | 54.451 | CXCL12 | Stromal cell-derived factor 1 | NM_000609 |
| VEGF R2 | 0.959 | 0.276 | 54.451 | CD309 antigen | Vascular endothelial growth factor receptor 2 | NP_002244.1. |
| Cardiotrophin-1 | 0.377 | 0.255 | 56.304 | CTF-1 | Cardiotrophin-1 | NM_001330 |
| GRO-alpha | 0.207 | 0.263 | 56.304 | CXCL1 | Growth-regulated alpha protein | NM_001511 |
| HCC-4 | 0.211 | 0.256 | 56.304 | CCL16 | Hemofiltrate CC chemokine 4 | NM_004590 |
| IGFBP-2 | 0.000 | 0.256 | 56.304 | IGFBP2 | Insulin-like growth factor binding proteins 2 | NM_000597 |
| MIG | 0.000 | 0.253 | 56.304 | CXCL9 | Chemokine (C-X-C motif) ligand 9 chemokine (C-X-C motif) ligand 9 | NM_002416.1 |
| Activin A | 0.354 | 0.232 | 57.427 | INHBA | Activin A | NM_002192 |
| BMP-7 | 0.371 | 0.232 | 57.427 | BMP7 | Bone morphogenetic protein 7 | NM_001719 |
| Fas Ligand | 0.409 | 0.240 | 57.427 | FASLG | Fas ligand | NM_000639 |
| GCP-2 | 0.000 | 0.236 | 57.427 | CXCL6 | Granulocyte chemotactic protein 2 | NM_002993 |
| IL-18 BPalpha | 0.440 | 0.232 | 57.427 | IL-18 BPa | Interleukin-18-binding protein | NP_001034748.1, |
| TGF-beta 3 | 0.132 | 0.241 | 57.427 | TGFB3 | Transforming growth factor beta-3 | NM_003239 |
| MCP-2 | 0.000 | 0.229 | 57.878 | CCL8 | C-C motif chemokine 8 | NM_005623 |
| EGF | 0.000 | 0.194 | 63.741 | EGF | Epidermal growth factor | NM_001963 |
| Endoglin | 0.396 | 0.176 | 63.741 | ENG | Endoglin | NM_000118 |
| FGF-6 | 0.000 | 0.186 | 63.741 | FGF6 | Fibroblast growth factor-6 | NM_020996 |
| FGF-9 | 0.190 | 0.163 | 63.741 | FGF9 | Fibroblast growth factor-9 | NM_002010 |
| IL-1 R II | 0.423 | 0.196 | 63.741 | IL-1 RII | Interleukin-1 receptor type II | NP_004624.1. |
| IL-13 Ralpha2 | 0.434 | 0.183 | 63.741 | IL13RA2 | Interleukin 13 receptor alpha 2 | NM_000640 |
| MIP-1alpha | 0.276 | 0.163 | 63.741 | CCL3 | C-C motif chemokine 3 | NM_002983 |
| MPIF-1 | 0.564 | 0.198 | 63.741 | CCL23 | Chemokine (C-C motif) ligand 23 | NM_005064.3 |
| MSP-alpha | 0.288 | 0.163 | 63.741 | MST1 | Macrophage stimulating 1 | NM_020998.3 |
| Osteoprotegerin | 0.295 | 0.163 | 63.741 | OPG | Tumor necrosis factor receptor superfamily member 11B | NM_002546 |
| PDGF Ralpha | 0.608 | 0.191 | 63.741 | PDGFRA | Alpha-type platelet-derived growth factor receptor | NM_006206 |
| PECAM-1 | 0.636 | 0.162 | 63.741 | PECAM-1 | Platelet endothelial cell adhesion molecule | NM_000442 |
| sTNF-RI | 0.314 | 0.169 | 63.741 | TNFRSF1A | Tumor necrosis factor receptor superfamily member 1A | NM_001065 |
| VEGF | 0.345 | 0.176 | 63.741 | VEGFA | Vascular endothelial growth factor A | NM_001025366 |
| VEGF-D | 0.349 | 0.195 | 63.741 | FIGF | Vascular endothelial growth factor D | NM_004469 |
| IP-10 | 0.487 | 0.149 | 64.943 | CXCL10 | Interferon-inducible protein-10 | NM_001565 |
| BMP-5 | 0.367 | 0.129 | 66.157 | BMP5 | Bone morphogenetic protein 5 | NM_021073 |
| IL-2 | 0.000 | 0.129 | 66.157 | IL2 | Interleukin 2 | NM_000586 |
| NGF R | 0.575 | 0.120 | 67.030 | NGFR | Tumor necrosis factor receptor superfamily member 16 | NM_002507 |
| I-TAC | 0.266 | 0.087 | 69.011 | CXCL11 | Interferon-inducible T cell alpha chemoattractant | NM_005409 |
| IL-8 | 0.262 | 0.088 | 69.011 | IL8 | Interleukin 8 | NM_000584 |
| AgRP | 0.148 | 0.000 | 70.115 | AGRP | Agouti related protein | NM_001138 |
| Amphiregulin | 0.155 | 0.000 | 70.115 | AREG | Amphiregulin | NM_001657 |
| b-NGF | 0.164 | 0.000 | 70.115 | NGFB | Nerve growth factor-beta | NM_002506 |
| bFGF | 0.161 | 0.000 | 70.115 | bFGF | Basic fibroblast growth factor | NP_001997.5 |
| BMP-4 | 0.000 | 0.000 | 70.115 | BMP4 | Bone morphogenetic protein 4 | NM_130850 |
| BTC | 0.167 | 0.000 | 70.115 | BTC | Betacellulin | NM_001729 |
| CCL-28 | 0.171 | 0.000 | 70.115 | CCL28 | C-C motif chemokine 28 | NM_148672 |
| CK beta 8-1 | 0.000 | 0.000 | 70.115 | CCL23 | Chemokine-beta-8 | NM_145898 |
| DR6 (TNFRSF21) | 0.391 | 0.000 | 70.115 | TNFRSF21 | Tumor necrosis factor receptor superfamily member 21 | NM_014452 |
| FGF-4 | 0.187 | 0.000 | 70.115 | FGF4 | Fibroblast growth factor-4 | NM_002007 |
| Fractalkine | 0.000 | 0.000 | 70.115 | CX3CL1 | Fractalkine | NP_002987.1 |
| GCSF | 0.194 | 0.000 | 70.115 | GCSF | Granulocyte-colony stimulating factor | NM_172220 |
| GITR | 0.200 | 0.000 | 70.115 | TNFRSF18 | Tumor necrosis factor receptor superfamily, member 18 | NM_004195.2 |
| GITR-Ligand | 0.197 | 0.000 | 70.115 | TNFSF18 | Tumor necrosis factor (ligand) superfamily, member 18 | NM_005092.3 |
| I-309 | 0.000 | 0.000 | 70.115 | CCL1 | T lymphocyte-secreted protein I-309 | NM_002981 |
| ICAM-3 | 0.221 | 0.000 | 70.115 | ICAM3 | Intercellular Adhesion Molecule 3 | NM_002162 |
| IGF-I | 0.000 | 0.000 | 70.115 | IGF1 | Insulin-like growth factor-1 | NM_000618 |
| IGF-I SR | 0.232 | 0.000 | 70.115 | IGF1R | Insulin-like growth factor 1 receptor | NP_000866.1 |
| IGFBP-3 | 0.225 | 0.000 | 70.115 | IGFBP3 | Insulin-like growth factor binding proteins 3 | NM_001013398 |
| IL-1 RI | 0.238 | 0.000 | 70.115 | IL-1 RI | Interleukin-1 receptor type I | NP_000868.1 |
| IL-10 Rbeta | 0.429 | 0.000 | 70.115 | IL10RB | Interleukin 10 receptor beta | NM_000628 |
| IL-11 | 0.242 | 0.000 | 70.115 | IL11 | Interleukin 11 | NM_000641 |
| IL-12 p40 | 0.246 | 0.000 | 70.115 | IL12B | Interleukin 12B | NM_002187.2 |
| IL-12 p70 | 0.249 | 0.000 | 70.115 | IL12A+IL12B | Interleukin 12 p70 | xxx |
| IL-17 | 0.253 | 0.000 | 70.115 | IL17A | Interleukin 17 p40 | NM_002190 |
| IL-18 Rbeta | 0.446 | 0.000 | 70.115 | IL18RAP | Interleukin-18 receptor accessory protein | NP_003844.1 |
| IL-1ra | 0.000 | 0.000 | 70.115 | IL1RA | Interleukin-1 receptor antagonist protein | NM_173842 |
| IL-2 Rapha | 0.256 | 0.000 | 70.115 | IL2RA | Interleukin-2 receptor alpha chain | NM_000417 |
| IL-2 Rbeta | 0.456 | 0.000 | 70.115 | IL2RB | Interleukin-2 receptor subunit beta | NM_000878 |
| IL-2 Rgamma | 0.462 | 0.000 | 70.115 | IL2RG | Cytokine receptor common gamma chain | NM_000206 |
| IL-21R | 0.468 | 0.000 | 70.115 | IL21R | Interleukin 21 receptor | NM_021798 |
| IL-5 Ralpha | 0.476 | 0.000 | 70.115 | IL5RA | Interleukin-5 receptor subunit alpha | NM_175724 |
| IL-9 | 0.481 | 0.000 | 70.115 | IL9 | Interleukin-9 | NM_000590 |
| Leptin R | 0.502 | 0.000 | 70.115 | LEPR | Leptin receptor | NM_002303 |
| LIF | 0.512 | 0.000 | 70.115 | LIF | Leukemia inhibitoty factor | NM_002309 |
| Lymphotactin | 0.269 | 0.000 | 70.115 | XCL1 | Lymphotactin | NM_002995 |
| MMP-13 | 0.543 | 0.000 | 70.115 | MMP13 | Collagenase 3 | NM_002427 |
| Oncostatin M | 0.299 | 0.000 | 70.115 | OSM | Oncostatin-M | NM_020530 |
| PIGF | 0.303 | 0.000 | 70.115 | PGF | Placenta growth factor | NM_002632 |
| SDF-1beta | 0.683 | 0.000 | 70.115 | CXCL12 | Stromal cell-derived factor 1 | NM_000609 |
| sTNF RII | 0.310 | 0.000 | 70.115 | TNFRSF1B | Tumor necrosis factor receptor superfamily member 1B | NM_001066 |
| TECK | 0.318 | 0.000 | 70.115 | CCL25 | Thymus-expressed chemokine | NM_005624 |
| TGF beta2 | 0.751 | 0.000 | 70.115 | TGFB2 | Transforming growth factor beta-2 | NM_003238 |
| Thrombopoietin | 0.329 | 0.000 | 70.115 | THPO | Thrombopoietin | NM_000460 |
| Tie-1 | 0.777 | 0.000 | 70.115 | Tie-1 | Tyrosine-protein kinase receptor Tie-1 | MGI:99906 |
| Tie-2 | 0.809 | 0.000 | 70.115 | TEK | Angiopoietin-1 receptor | NM_000459 |
| VE-Cadherin | 0.893 | 0.000 | 70.115 | CDH5 | Vascular endothelial cadherin | NM_001795 |
