# Supplementary material for: An antibody microarray analysis of serum cytokines in neurodegenerative Parkinsonian syndromes
Source: Proteome Sci. 2012 Nov 23;10:71. doi: 10.1186/1477-5956-10-71 (PMC3539904; doi:10.1186/1477-5956-10-71)

Additional file 3

Differential expression of PDGF-BB and Prolactin in patients with PSP/CBS, MSA and controls without anti-Parkinsonian treatment.

Individual data points are shown as circles and horizontal bars indicate medians. In addition data are shown as box plot with medians indicated as horizontal bars with boxes. Groups were compared using the Kruskal-Wallis test and Dunn's multiple comparison post-hoc test and overall p-values are shown in each figure.

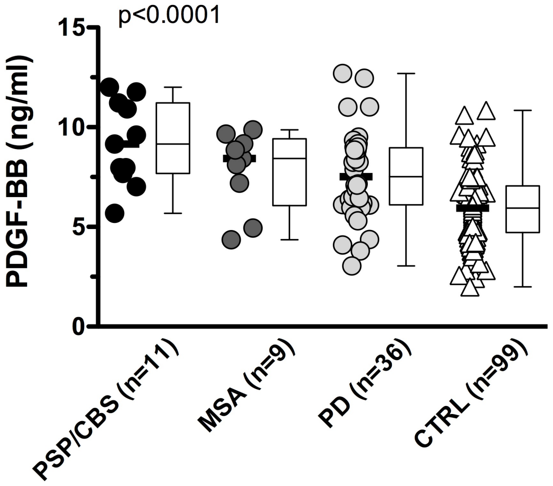

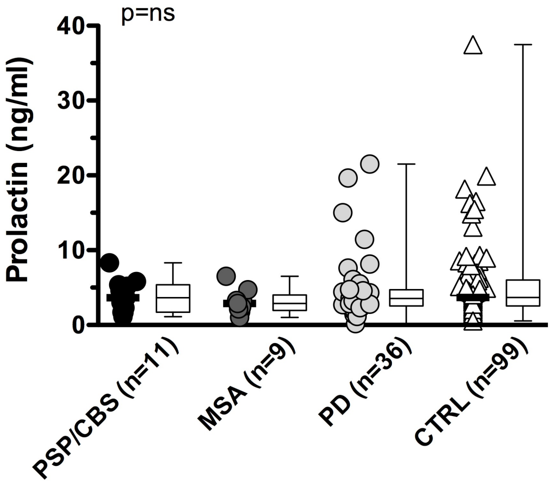

Supplement: Additional file 2 — Differential expression of PDGF-BB and prolactin in patients with PSP/CBS, MSA and controls without anti-Parkinsonian treatment. [file 1477-5956-10-71-S2.pdf]
