# Supplementary material for: An antibody microarray analysis of serum cytokines in neurodegenerative Parkinsonian syndromes
Source: Proteome Sci. 2012 Nov 23;10:71. doi: 10.1186/1477-5956-10-71 (PMC3539904; doi:10.1186/1477-5956-10-71)
Supplement: Additional file 3 — Technical characteristics of the immunoassays used for validation. [file 1477-5956-10-71-S3.docx]

### Additional file 1

### Technical characteristics of the immunoassays used for validation

| **Cytokine** | **Order Number** | **Company** | **Detection**  **limit** | **Standard**  **curve** | **Sample**  **dilution** | **Intra-assay variation** | **Inter-assay variation** | **Specificity** |
| --- | --- | --- | --- | --- | --- | --- | --- | --- |
| **Fluorescence bead-based assays (Flow Cytomix)** | | | | | | | | |
| sICAM-1 | BMS80201FF | eBioscience | 5.3 ng/ml | 0-4000 ng/ml | undiluted | 2.9% | 2.0% | 100% |
| Leptin | BMS82039/2FF | eBioscience | 0.05 ng/ml | 0-250 ng/ml | undiluted | 2.7% | 8.2% | 100% |
| PDGF-BB | BMS82071FF | eBioscience | 3.4 pg/ml | 0-15000 pg/ml | undiluted | 7.6% | 6.1% | 100% |
| **ELISA** | | | | | | | | |
| IL-2RA | BMS212INSTCE | eBioscience | 0.2 ng/ml | 0-20 ng/ml | 1:15 | 7.7% | 10.0% | 100% |
| RANTES | BMS287/2INST | eBioscience | 4.2 pg/ml | 0-2000 pg/ml | 1:15 | 6.9% | 9.9% | 100% |
| MCP-4 | DY327 | R&D Systems | 11.4 ng/ml | 0-500 ng/ml | 1:2 | 9.5% | 11.0% | 99.8% |
| Prolactin | DY682 | R&D Systems | 11.0 pg/ml | 0-1000 pg/ml | 1:10 | 8.3% | 9.0% | 100% |

All data were provided by the manufacturers, except for MCP-4 and Prolactin, this data were determined in our laboratory.
